# Do Not Dismiss Incidental Hyperkalemia in Childhood: Early Recognition of Pseudohypoaldosteronism Type II

**DOI:** 10.7759/cureus.103496

**Published:** 2026-02-12

**Authors:** Hiroaki Kanai, Hiroki Sato

**Affiliations:** 1 Department of Pediatrics, Suwa Central Hospital, Chino, JPN; 2 Department of Pediatrics, Faculty of Medicine, University of Yamanashi, Chuo, JPN

**Keywords:** family history, hyperkalemia, metabolic acidosis, pseudohypoaldosteronism type ii, urinary potassium excretion

## Abstract

Persistent hyperkalemia in children warrants careful evaluation, as it may indicate an underlying renal tubular disorder. We report a case of persistent hyperkalemia in a three-year-old boy in which adenovirus infection unmasked latent pseudohypoaldosteronism type II (PHAII), allowing recognition prior to the development of hypertension. The patient presented with a nine-day history of fever. Initial laboratory tests showed hyperkalemia (5.8 mmol/L), mild hyponatremia, normal renal function, and normal anion gap (AG) metabolic acidosis (pH: 7.37; bicarbonate: 19.9 mmol/L; AG: 8.1 mmol/L). The patient was diagnosed with adenovirus infection. Although the fever and inflammatory markers improved within four days, the hyperkalemia persisted (6.2 mmol/L). At three and six weeks, serum potassium remained elevated (6.0 and 6.4 mmol/L, respectively) with normal AG metabolic acidosis (HCO₃⁻ 20.0 and 17.6 mmol/L, respectively; AG 12.8 and 11.4 mmol/L, respectively). Urinary potassium indices showed inappropriately low fractional excretion of potassium (FEK) and transtubular potassium gradient (TTKG) with normal renin-aldosterone levels. Subsequently, family history-taking identified early-onset hypertension in the child’s father. Treatment with hydrochlorothiazide (HCTZ) resulted in normalization of serum potassium and correction of the metabolic acidosis. Genetic testing revealed a heterozygous *KLHL3* variant (c.1501C>T, p.Pro501Ser), confirming the diagnosis of PHAII. This case highlights the importance of not dismissing incidentally detected hyperkalemia in children and illustrates the value of performing a structured evaluation that includes confirming the persistence of hyperkalemia, acid-base assessment, urinary potassium analysis, and family history-taking to facilitate early diagnosis of renal tubular disorders such as PHAII.

## Introduction

Hyperkalemia can result from reduced renal potassium excretion, excessive potassium intake, medication effects, or a transcellular shift due to acidosis, diabetes mellitus, or massive tissue breakdown, as seen in rhabdomyolysis [1,2]. Spurious hyperkalemia is common in pediatric practice, often due to hemolysis during blood sampling; however, true hyperkalemia in children is rare [3]. Consequently, incidental findings are often dismissed as transient or artifactual [2]. However, persistent hyperkalemia warrants systematic evaluation, as it may indicate an underlying renal tubular disorder.

Pseudohypoaldosteronism type II (PHAII) is a monogenic disorder characterized by hypertension, hyperkalemia, and metabolic acidosis with a normal anion gap (AG) [3,4]. It involves sodium-chloride cotransporter (NCC) activity in the distal convoluted tubule [4]. NCC overactivity enhances sodium and chloride reabsorption [5]. The consequent impaired delivery of sodium to the collecting duct results in impaired aldosterone-mediated exchange of sodium for potassium and protons, resulting in hyperkalemia and hyperchloremic metabolic acidosis, despite normal aldosterone production [5]. This disease is considered to be rare; however, the true incidence is unknown because the available evidence is limited to case reports and small case series, with no population-based studies. It is likely underdiagnosed, not only in children but also in adults with hypertension, as genetic testing is not routinely performed. Although hypertension usually develops in late childhood or even adulthood, the metabolic disorders, hyperkalemia and metabolic acidosis, can precede hypertension and may be present at birth [5,6]. Therefore, PHAII should be considered in the differential diagnosis of unexplained hyperkalemia in children of all ages, even if they are normotensive. Here, we report a pediatric case of hyperkalemia in which adenovirus infection unmasked previously latent PHAII, allowing recognition of the disease in an early stage prior to the onset of hypertension.

## Case presentation

A previously healthy three-year-old boy with normal growth and development presented with a nine-day history of fever. Initial laboratory test results revealed hyperkalemia and low sodium levels, with chloride and creatinine levels within normal limits. Venous blood gas analysis was consistent with metabolic acidosis with partial respiratory compensation. The white blood cell (WBC) count and C-reactive protein (CRP) level were elevated, indicating acute inflammation (Table 1). A respiratory panel confirmed adenovirus infection.

**Table 1:** The patient’s serial blood test results AG: anion gap; Cl: chloride; CRP: C-reactive protein; HCO₃⁻: bicarbonate; HCTZ: hydrochlorothiazide; K: potassium; n.a.: not available; Na: sodium; PHAⅡ: pseudohypoaldosteronism type II; WBC: white blood cell ^a^HCTZ was initiated at a dose of 0.7 mg/kg/day at this visit; ^b^The HCTZ dose was increased to 1.5 mg/kg/day at this visit

Variable	On presentation	Four days after presentation	Three weeks after presentation	Six weeks after presentation^a^	One month after HCTZ initiation	Three months after HTCZ initiation^b^	One month after HCTZ dose escalation	Reference value
WBC count (× 10^3^ cells/µL)	14.7	11.9	9.9	8.6	-	-	-	3.9-9.8
CRP (mg/dL)	5.99	1.62	0.34	0.08	-	-	-	<0.15
Creatinine (mg/dL)	0.25	0.30	0.34	-	-	-	-	0.21-0.37
Na (mmol/L)	132.7	137.0	137.0	137.1	139.5	139.5	136.9	138-145
K (mmol/L)	5.8	6.2	6.0	6.4	4.7	5.8	4.6	3.6-4.8
Cl (mmol/L)	104	105	108	109	105	110	105	101-108
pH	7.37	n.a.	7.31	7.28	7.36	7.33	7.38	7.32-7.41
PCO_2_ (mmHg)	34.9	n.a.	40.9	38.1	46.2	37.8	42.3	42-53
HCO₃⁻ (mmol/L)	19.9	n.a.	20.0	17.6	25.2	19.6	24.6	24-28
AG (mmol/L)	8.1	n.a.	12.8	11.4	12.4	11.4	10.3	7-13
Plasma renin activity (ng/mL/h)	n.a.	n.a.	n.a.	0.6	-	-	-	0.2-2.3
Aldosterone (pg/mL)	n.a.	n.a.	n.a.	38.0	-	-	-	29.9-158.8

Four days later, the fever had resolved, and the WBC count and CRP level had decreased; however, the hyperkalemia persisted. Three weeks after presentation, the patient was re-evaluated for persistent hyperkalemia. His height was 99.0 cm, weight was 16.2 kg, and blood pressure (96/55 mmHg) was normal for a child of his age. Blood tests revealed persistent hyperkalemia with normal AG metabolic acidosis (Table 1). The urinary indices suggested reduced renal potassium excretion, with a reduced fractional excretion of potassium (FEK) and transtubular potassium gradient (TTKG) (Table 2). At this time, the urinary sodium concentration was high, and the urine osmolality exceeded the plasma osmolality (Table 2), supporting a valid interpretation of TTKG [7]. Six weeks after presentation, the hyperkalemia with normal AG metabolic acidosis persisted (Table 1), with reduced FEK and TTKG (Table 2). Again, the urinary sodium concentration was high, and the urine osmolality exceeded the plasma osmolality (Table 2); however, the plasma renin activity and aldosterone levels were within normal limits (Table 1). Renal ultrasonography showed no abnormalities. At this time, the patient’s father reported a history of hypertension, having been diagnosed in his twenties.

**Table 2:** Assessment of the patient’s urinary potassium excretion and related measures FEK: fractional excretion of potassium; n.a.: not available; Na: sodium; TTKG: transtubular potassium gradient ^a^The prerequisites for measuring TTKG, namely, urine osmolality greater than plasma osmolality and urinary sodium >25 mmol/L, were met

Variable	Three weeks after presentation	Six weeks after presentation	Reference value
FEK (%)	7.6	9.1	>20% during hyperkalemia
TTKG^a^	2.9	5.3	>6 during hyperkalemia
Urinary Na (mmol/L)	146.3	212.9	n.a.
Urine osmolality (mOsm/kg)	1181	921	200-800
Plasma osmolality (mOsm/kg)	286	291	275-285

The constellation of persistent hyperkalemia, normal AG metabolic acidosis, reduced renal potassium excretion, and a family history of early-onset hypertension suggested PHAII. Treatment with hydrochlorothiazide (HCTZ) was initiated at a dose of 0.7 mg/kg/day, and on retesting one month later, the potassium and bicarbonate levels were within the reference range. However, retesting two months later (three months after initiating HCTZ treatment) revealed a recurrence of hyperkalemia and normal AG metabolic acidosis. The dose of HCTZ was increased to 1.5 mg/kg/day, and on retesting one month later, the potassium and bicarbonate levels were again within the reference range. The serial laboratory findings during the clinical course are summarized in Table 1.

**Figure 1:**
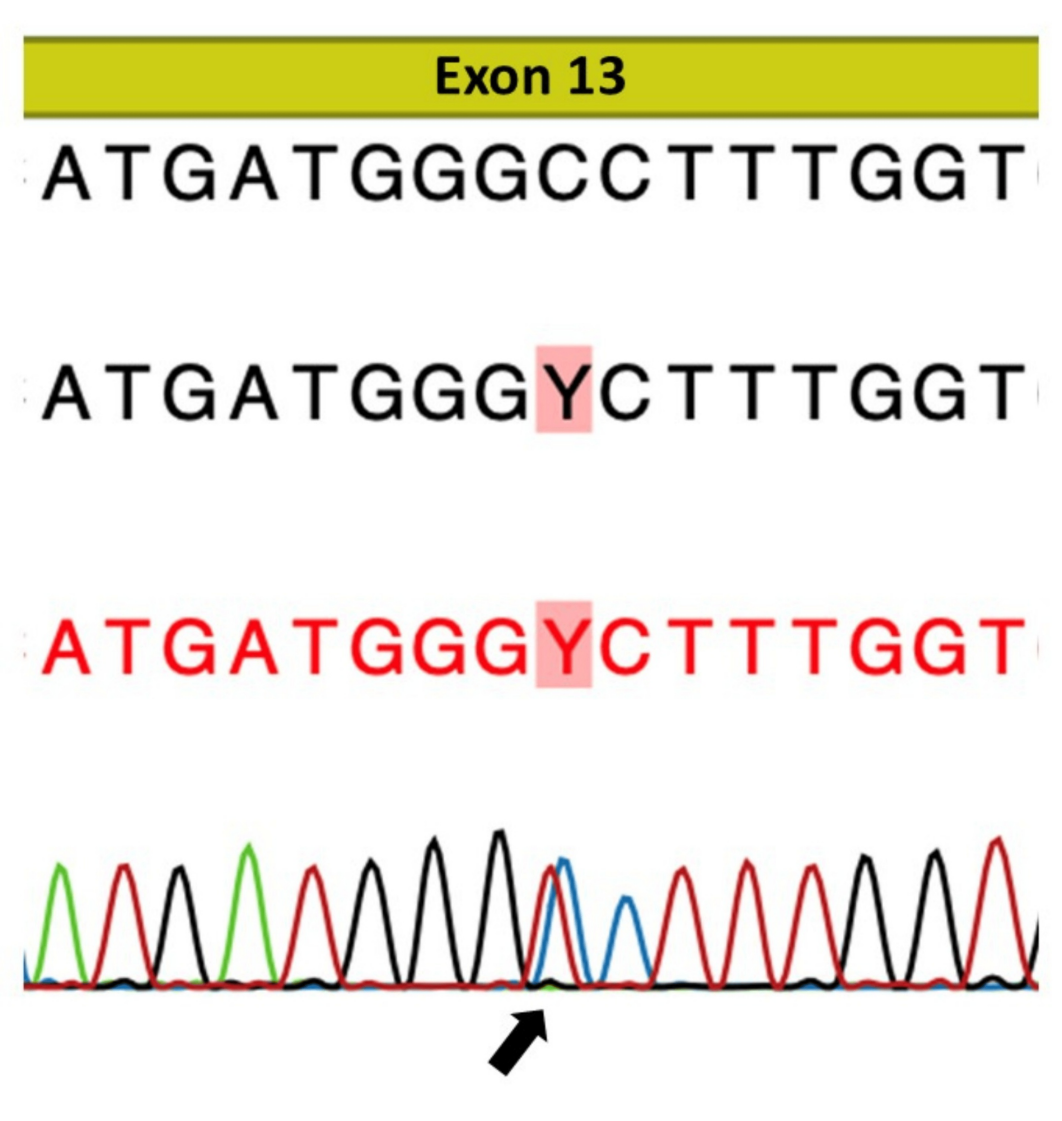
Gene sequencing results The figure shows a heterozygous C-to-T substitution at nucleotide position c.1501 in exon 13 (c.1501C>T; arrow), resulting in a heterozygous missense mutation. The change involves a substitution of proline to serine at position 501 (p.Pro501Ser)

Gene sequence analysis identified a heterozygous missense mutation in *KLHL3* (c.1501C>T, p.Pro501Ser, Exon 13) (Figure 1), listed in the Human Gene Mutation Database (ID: 22266938) and classified as a variant of uncertain significance. However, considering the biochemical phenotype and clear responsiveness to thiazide therapy, we judged this variant to be pathogenic.

## Discussion

PHAII, also known as Gordon syndrome, is a renal tubular disorder caused by increased NCC activity in the distal convoluted tubule, leading to increased sodium reabsorption and decreased potassium and hydrogen excretion [5]. Causative mutations involve WNK1, *WNK4*, *KLHL3*, and *CUL3* [6]. *KLHL3* and *CUL3* were first recognized as causative genes in 2012 [8]. Most cases follow an autosomal dominant inheritance pattern, although autosomal recessive *KLHL3* variants have been reported [4]. Phenotypic severity varies by genotype, ranked in decreasing order as follows: *CUL3*, recessive *KLHL3*, dominant *KLHL3*, *WNK4*, and *WNK1* [6,8]. PHAII responds well to treatment with thiazide diuretics, which directly inhibit NCC, correcting the electrolyte imbalance and preventing hypertension.

A PubMed search of PHAII cases in children aged under 15 years, published since 2012 (when *KLHL3* and *CUL3* gene variants were recognized as causative), identified case reports in which the reason for the blood tests was explicitly reported (Table 3) [3,5,9-19]. Almost all cases were detected incidentally, typically during evaluations of unrelated conditions such as acute illness, poor growth or feeding, preoperative testing, or routine laboratory screening. The PubMed search identified only one child with PHAII who was diagnosed specifically as a result of investigating unexplained hypertension. However, despite PHAII being discovered incidentally, most children in published pediatric case reports already exhibited hypertension. *KLHL3* and *CUL3* variants predominated among the reported cases, reflecting the earlier and more severe phenotypes associated with variants in these genes. Our patient presented with fever due to an adenovirus infection, initially raising the question of whether acute illness or dehydration contributed to the electrolyte abnormalities. However, the persistent hyperkalemia and metabolic acidosis after complete recovery indicate that the infection was not the primary cause. Instead, the acute illness prompted the laboratory testing that led to the diagnosis. This case differs from most previously reported cases in that hypertension was absent, representing an early biochemical stage of PHAII and providing insight into early disease evolution.

**Table 3:** Clinical features and laboratory findings of published cases of pediatric pseudohypoaldosteronism type Ⅱ HCO₃⁻: bicarbonate; K: potassium; n.a.: not available; +: present; −: absent

Reference	Age	Reason for the blood tests	K (mmol/L)	HCO3^-^ (mmol/L)	Hypertension	Mutated gene
Tsuji et al., 2013 [9]	3 years	Croup	6.8	12.6	+	CUL3
Mitani et al., 2016 [10]	10 months	Loss of consciousness	6.6	14.8	+	KLHL3
Hollander et al., 2016 [3]	6 years	Hematuria	8.6	16	+	CUL3
Hollander et al., 2016 [3]	4 months	Decrease in enteral feeding	7.0	22	+	KLHL3
Park et al., 2017 [11]	9 months	Urinary tract infection	6.3	13	+	KLHL3
Park et al., 2017 [11]	20 months	Intussusception	6.9	12	+	KLHL3
Doan et al., 2020 [12]	2 months	Feeding difficulty	6.6	n.a.	n.a.	KLHL3
Yavas Abali et al., 2020 [13]	14 years	Short stature, excessive weight gain	6.4	n.a.	+	CUL3
Nakano et al., 2020 [14]	2 months	Respiratory syncytial virus infection	n.a.	n.a.	n.a.	CUL3
Ostrosky-Frid et al., 2020 [15]	12 years	Hypertension	7.0	17.8	+	CUL3
Patti et al., 2021 [16]	19 months	Fever	7.45	15.2	+	CUL3
Park et al., 2022 [17]	7 years	Short stature	6.8	16.7	+	CUL3
Babar et al., 2022 [18]	5 weeks	Difficulty in feeding, lethargy, emesis	10.1	n.a.	−	WNK1
Zieg et al., 2025 [5]	3 years	Preoperative work-up	7.6	11.3	+	KLHL3
Zieg et al., 2025 [5]	10 days	Insufficient weight gain	7.4	16	n.a.	KLHL3
Modi et al., 2025 [19]	14 years	Abdominal pain, vomiting, watery diarrhea	5.6	19	−	WNK1

In this case, persistent hyperkalemia despite preserved glomerular function, accompanied by normal AG metabolic acidosis, led us to conduct further investigations. These revealed impaired renal potassium excretion (low FEK and TTKG values), which led us to suspect PHAII. This case offers several important educational points. First, incidental hyperkalemia in children should not be dismissed without confirmation. Although pseudohyperkalemia is common in children, clinicians should not attribute it solely to transient or artifactual causes, and repeated testing to verify persistent hyperkalemia is a crucial first step to diagnosis, even in mild or asymptomatic hyperkalemia. In addition, clinicians should also assess the acid-base balance carefully because concomitant normal AG metabolic acidosis is an important clue to diagnosis and suggests a renal tubular disorder rather than a systemic cause. Second, urinary potassium excretion should be assessed. Urinary potassium indices are simple, but essential diagnostic markers. Persistently low FEK and TTKG values during hyperkalemia indicate impaired renal tubular excretion [1]. The FEK values provide evidence of impaired renal potassium handling. Additionally, the TTKG, which reflects aldosterone-dependent potassium secretion in the distal nephron, is particularly informative when interpreted under appropriate physiological circumstances, specifically, urinary sodium concentration >25 mmol/L and urine osmolality greater than plasma osmolality [7]. In this case, persistently low FEK and TTKG values during hyperkalemia, combined with urinary sodium concentrations >25 mmol/L and urine osmolality greater than plasma osmolality at both urinary assessments, provided strong evidence of inappropriate low urinary potassium secretion, despite persistent hyperkalemia. Finally, given the autosomal dominant inheritance pattern in most PHAII cases, a family history of early-onset hypertension is an important diagnostic clue, even when the child is normotensive [8]. In this case, the father’s early-onset hypertension, diagnosed in his twenties, was only identified at the patient’s fourth visit, suggesting that earlier inquiry into family history might have expedited diagnosis. This highlights the importance of obtaining a detailed family history during the initial evaluation of persistent hyperkalemia. Greater awareness of these principles may facilitate earlier detection of PHAII, enabling timely treatment and preventing progression to hypertension and its complications.

This report has some limitations. It describes a single case, so the generalizability of our findings may be limited. In addition, the identified *KLHL3* variant is classified as a variant of uncertain significance, although the clinical phenotype and therapeutic response to thiazide diuretics in this case strongly support its pathogenicity.

## Conclusions

This case highlights the importance of not dismissing incidentally detected hyperkalemia in children and illustrates the value of using a structured, stepwise approach to the investigation and management of pediatric hyperkalemia, and of confirming its persistence through repeated measurement. If the hyperkalemia persists, acid-base balance (pH and bicarbonate level) and renal potassium excretion should be assessed, and inquiries should be made about a possible family history of early-onset hypertension. This structured approach can identify renal tubular disorders such as PHAII even in apparently healthy children, preventing diagnostic delays and ensuring timely, effective management.
